# Genome-wide analysis of transposable elements and tandem repeats in the compact placozoan genome

**DOI:** 10.1186/1745-6150-5-18

**Published:** 2010-04-15

**Authors:** Shi Wang, Lingling Zhang, Eli Meyer, Zhenmin Bao

**Affiliations:** 1 Section of Integrative Biology, School of Biological Sciences, University of Texas at Austin, 1 University Station C0930, Austin, TX 78712, USA; 2 Waggoner Center for Alcohol and Addiction Research, University of Texas at Austin, 1 University Station A4800, Austin, TX 78712, USA; 3 Key Laboratory of Marine Genetics and Breeding, Ocean University of China,5 Yushan Road, Qingdao 266003, China

## Abstract

The placozoan *Trichoplax adhaerens *has a compact genome with many primitive eumetazoan characteristics. In order to gain a better understanding of its genome architecture, we conducted a detailed analysis of repeat content in this genome. The transposable element (TE) content is lower than that of other metazoans, and the few TEs present in the genome appear to be inactive. A new phylogenetic clade of the *gypsy*-like LTR retrotransposons was identified, which includes the majority of *gypsy*-like elements in *Trichoplax*. A particular microsatellite motif (ACAGT) exhibits unexpectedly high abundance, and also has strong association with its nearby genes.

**Reviewers:**

This article was reviewed by Dr. Jerzy Jurka and Dr. I. King Jordan.

## Findings

Placozoans are arguably the simplest free-living multicelluar animals, and may represent an extant example of the ancestral metazoan body plan [1]. A recent comprehensive phylogenetic study suggests that placozoans are basal relatives to all other non-Bilaterian animals [[2], but see [3]]. It has been suggested that the placozoan *Trichoplax adhaerens *is an excellent model for the study of early evolution of metazoans [4,5]. The recent analysis of the *Trichoplax *genome has revealed a lack of the frequent intron loss and genomic rearrangement that characterize other small metazoan genomes (e.g. flies and worms), and many structural aspects (e.g. introns, local gene order and larger-scale linkages) are thought to represent ancestral eumetazoan characteristics [1]. In order to gain a better understanding of the evolution of the *Trichoplax *genome architecture, it may be interesting to investigate the abundance and types of repetitive sequences in the *Trichoplax *genome, because repetitive sequences, especially transposable elements (TEs), are major evolutionary contributors that drive genome evolution by enhancing genome plasticity [6-8].

We adopted both homology-based and *ab initio *methods to search for putative TEs. A full description of these methods is available in additional file 1. The ~95 Mb *Trichoplax *genome sequence was compared with the Repbase database 14.04 [9] using tblastx [10] with an e-value threshold of 10^-4^, revealing 139 putative elements with significant similarity to known TEs (available in additional file 2). These elements accounted for only 0.13% of the *Trichoplax *genome, which is much lower than TE content of other small metazoan genomes (Table 1). The low TE content of the *Trichoplax *genome reported here is consistent with the previous report [1], in which 665 putative TEs were identified using the RepeatMasker program although no data curation and TE characterization were performed. The scarcity of TEs may explain why this genome has undergone the fewest rearrangement among metazoan genomes [1], because TEs are generally believed to be the major facilitators of this process [6-8]. The putative TEs we identified included representatives of three major TE classes: long terminal repeat (LTR) retrotransposons, non-LTR retrotransposons and DNA transposons (Table 2). DNA transposons were the most abundant TEs in the *Trichoplax *genome, and included diverse superfamilies such as *helitron*, *piggyBac*, *hAT*, *mariner/Tc1*, *polinton *and *MuDR*. LTR retrotransposons were the second most abundant TEs in the *Trichoplax *genome, and the majority of these belonged to the *gypsy *superfamily. Non-LTR retrotransposons were rare in the *Trichoplax *genome, and were represented by only three superfamilies. Most of the elements we identified contained frequent stop codons or/and frameshifts in the coding regions, and appeared fragmented. Putative complete open reading frames (ORFs) were identified in only 6 elements (additional file 2), of which half belonged to *hAT*, and half to *mariner*/*Tc1*. However, further investigation revealed that these lacked terminal inverted repeats (TIRs), and that each was present as only a single copy in the genome (blastn, e ≤ 10^-4^). Overall, none of the elements we identified contain all the features required for functional activity in the *Trichoplax *genome, a finding that is also supported by the complete lack of putative TEs in the 14,572 expressed sequence tags (ESTs) available for *T. adhaerens *(tblastx against the Repbase database 14.04, e ≤ 10^-4^). In an effort to identify novel TEs in the *Trichoplax *genome, we conducted searches for LTR retrotransposons and miniature inverted-repeat transposable elements (MITEs) based on their respective structural features. Ten putative LTR retrotransposons were identified using the LTR_FINDER program [11], and none of them showed protein homology to known LTR retrotransposons or other TEs. Moreover, none of them were present in the genome with more than two copies, so it's unlikely that these are true LTR retrotransposons. MITEs belong to nonautonomous DNA transposons, and are usually present in eukaryotic genomes in very high copy numbers [12]. MITE analysis was carried out using the MUST program [13]. We considered a potential MITE family by requiring at least 3 elements in this family with the same perfect TIRs and target site duplications (TSDs). MITEs turned out to be quite rare in the genome, with only a single family represented by ~20 copies altogether. This is anticipated since no functional autonomous TEs, upon which MITEs rely for their transposition and persistence, were identified in the *Trichoplax *genome. Indeed, in a search of *Trichoplax *TEs in the Repbase database [9], only five putative nonautonomous DNA transposons with low copy numbers have been reported so far [14].

**Table 1:** Summary of repeat content in the placozoan and other metazoan genomes.

	Placozoan (*T. adhaerens*)	Nematode (*C. elegans*)	Arthropod (*D. melanogaster*)	Chordate (*T. nigroviridis*)	Chordate (*H. sapiens*)
Genome size (Mb)	104	97	180	340	3,200

Gene no.	~11,500	~19,000	~13,600	~22,400	~31,000

**Transposable element (TE)**	0.13%	6.5%	3.1%	0.9%	44.4%

LTR retrotransposon	0.04%	0.0%	1.5%	0.1%	8.1%

Non-LTR retrotransposon	0.003%	0.4%	0.7%	0.8%	33.4%

DNA transposon	0.09%	5.3%	0.7%	0.0%	2.8%

Active TE	None	Yes	Yes	Yes	Yes

**Tandem repeat**	2.7%	2.7%	3%	4.5%	3%

Microsatellite	0.2%	0.2%	0.5%	3.2%	1.5%

Major SSR motif	ACAGT	AG	AC	A	AC

Minisatellite/Satellite	2.4%	2.5%	2.5%	1.3%	1.5%

Reference	[1]	[18,24,25]	[18,25-28]	[29-31]	[25]

**Table 2:** Classification of transposable elements (TEs) in the *Trichoplax *genome.

TE Superfamily	Counts	Matching Length (bp)
***LTR Retrotransposon***		

Gypsy	53	38727

BEL	1	903

DIRS	1	633

ERV1	1	480

***Non-LTR Retrotransposon***		

R4	1	1395

Jockey	1	867

Penelope	1	429

***DNA Transposon***		

Helitron	17	12820

PiggyBac	16	14676

hAT	14	16035

Mariner/Tc1	11	9098

Polinton	10	14820

MuDR	6	3498

Harbinger/PIF	3	2479

ISL2EU	2	6071

Chapaev	1	2036

**Total**	139	124967

**Figure 1:**
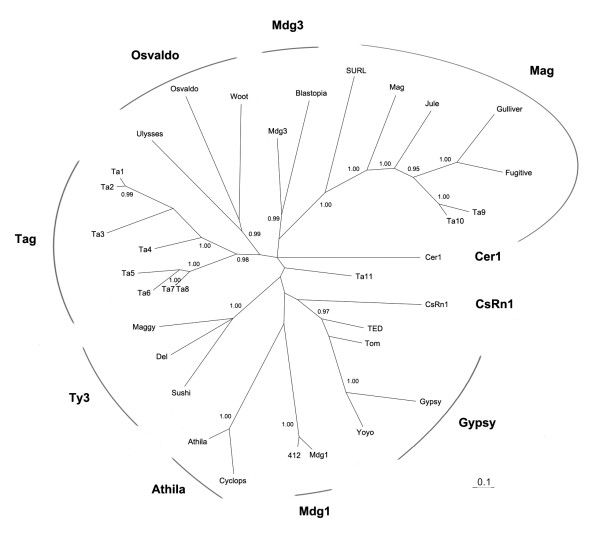
**Phylogenetic relationships of *T. adhaerens gypsy*-like elements (*Ta1-11*) and other elements from the *gypsy *group based on a Bayesian analysis of reverse transcriptase (RT) protein sequences**. Posterior probability exceeding 0.90 are shown. The sequences of most elements are derived from the EMBL online database [EMBL:DS36733] [15], while the others are as follows: *CsRn1 *[GenBank:AAK07487], *Gulliver *[GenBank:AF243513], *Fugitive *[GenBank:BK005226] and *Jule *[GenBank:AF278691]. RT sequences were aligned using the ClustalW method [32]. The protein alignment is available in the additional file 3. Phylogenetic analysis was performed with the program MrBayes 3.1 [33]. The appropriate model of evolution was identified as WAG+G+I [34] using the MCMC model-jumping method. The MCMC chain was run for 1,000,000 generations with a sample frequency of 200. In total, 5000 trees were produced, of which the first 4500 were discarded as burn-in while summarizing the data.

Further investigation of phylogenetic relationships within each TE superfamiliy was generally hampered by the low abundance and degenerated state of TEs representing the identified TE superfamilies. However, eleven *gypsy*-like elements (*Ta1-11*) shared a recognizable reverse transcriptase (RT) region, thus enabling investigation of their phylogenetic relationships. No outgroup was included in the phylogenetic analysis because we could not obtain a well-defined phylogeny based on the limited RT protein sequences when outgroup sequences were added. To date, nine phylogenetic clades have been identified in the *gypsy *group [15,16]. Our phylogenetic analysis of placozoan *gypsy*-like elements revealed a new clade (named *Tag*), which was formed by eight elements, *Ta1-8 *(Fig. 1, additional file 3). Since most of placozoan *gypsy*-like elements belong to the *Tag *clade, and this new clade has not been identified before in other metazoan genomes, this may suggest that much of the diversity among *gypsy*-like clades emerged after the divergence of *Trichoplax *from other metazoan lineages. It is also possible that *Tag *clade may represent an ancestral *gypsy *clade which was still preserved in the *Trichoplax *genome. Further identification and characterization of full-length elements belonging to the *Tag *clade from other eukaryotic organisms may provide new insights into the origin and diversification of the *gypsy*-like LTR retrotransposons. Two elements (*Ta9 *and *Ta10*) were grouped into the previously identified *Mag *clade [15]. The element *Ta11 *may represent another new clade, but more data from other animal genomes are needed to verify this.

**Figure 2:**
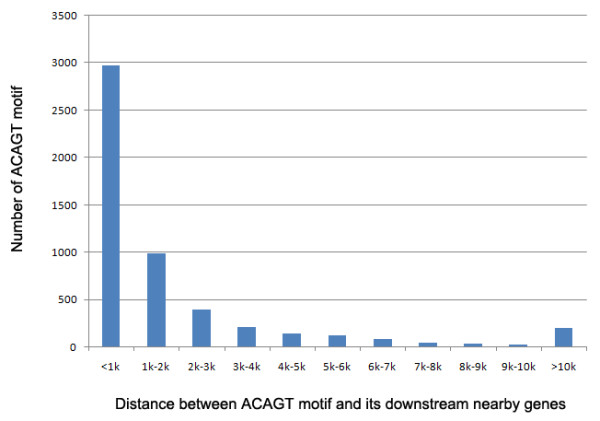
**Association pattern between ACAGT motif and its downstream nearby genes**.

In contrast to the extremely low TE content, tandem repeats including microsatellites and minisatellites, represented a major source of repeat sequences, and accounted for 2.7% of the *Trichoplax *genome. The tandem repeat content of the *Trichoplax *genome is thus comparable to those of other small metazoan genomes, and even the huge human genome (Table 1). Using the SciRoko program [17], 11,697 microsatellites (repeat units within 1~6 bp) were identified in the *Trichoplax *genome based on criteria described in a previous study (perfect repeats > 12 bp long), which had surveyed and analyzed the abundance and distribution of microsatellites in diverse eukaryotic taxonomic groups [18]. Surprisingly, we found that pentanucleotides were the most abundant repeat type in the *Trichoplax *genome, and accounted for 61.8% (i.e. 7233) of total microsatellites (additional file 4). This is unusual because mono-, di- and trinucleotides are usually the most abundant repeat types of microsatellites investigated in eukaryotic genomes so far [18-21]. To our knowledge, this finding represents the first report of pentanucleotide as the most abundant repeat type of microsatellites in a eukaryotic genome. More interestingly, further investigation revealed that majority (78%) of the pentanucleotide repeats were accounted for by a single motif (ACAGT). If this particular motif is discounted, other pentanucleotides only account for 13.8% of total microsatellites, which is slightly lower than the abundance (14.2%) of dinucleotides, suggesting the unusual pentanucleotide abundance in the *Trichoplax *genome is driven by the highly abundant ACAGT motif. Investigation of microsatellite abundance in the *Trichoplax *EST sequences revealed that as expected, trinucleotides, other than pentanucleotides, were the most abundant repeat type (52.4%), although ACAGT motif was still the most abundant pentanucleotide motif. Many studies have shown that microsatellites can serve as transcription factor binding sites (TFBSs) to regulate gene expression [for a review, see [19]]. In order to evaluate if ACAGT motif could be a potential TFBS, we investigated the association pattern of ACAGT motif and its downstream nearby genes (Fig. 2, more info in additional file 5). Strikingly, 54% and 85% of ACAGT motif located within 1 kb and 5 kb upstream of nearby genes respectively, suggesting the potential role of ACAGT motif in regulation of nearby gene expression. Further gene ontology (GO) enrichment analysis of ACAGT associated genes (GO term level = 6 and distance threshold = 5 kb) revealed that 58 genes significantly enriched in the GO term of translation (adjusted *p *< 0.021), and 155 in the protein modification process (adjusted *p *< 0.0064). Most of the translation genes are ribosomal proteins, and most of the protein modification genes are kinases. Kinases are known to regulate the majority of cellular pathways, especially those involved in signal transduction. Previous study has shown that *Trichoplax *genome encodes a rich array of transcription factors and signaling pathways that are typically associated with eumetazoan developmental patterning and cell-type specification [1]. It would therefore be interesting to explore the potential roles of ACAGT motif in these biological processes in the future.

Minisatellites (repeat units usually within 7~2000 bp) were detected using the program Tandem Repeat Finder 4.03 [22]. In total, 9208 minisatellites with repeat units ranging from 7 bp to 1204 bp were identified in the *Trichoplax *genome, and accounted for 2.4% of the genome size. In general, the smaller the repeat unit, the higher the repeat abundance (additional file 6a). Minisatellites with repeat units ranging from 7 bp to 25 bp contained highly abundant of repeats (usually >100 repeats). Similar distribution patterns have previously been observed in a scallop genome [23]. The average copy number of these repeats was generally low in the *Trichoplax *genome (< 4 copies for 94% of minisatellite repeat types) (additional file 6b), which may also account for the lack of frequent genomic rearrangements in the *Trichoplax *genome.

In summary, we conducted a detailed analysis of repeat content in the *Trichoplax *genome. The TEs in the *Trichoplax *genome are scarce and apparently lack functional activity. A new phylogenetic clade (*Tag*) of the *gypsy*-like LTR retrotransposons was identified. The unexpectedly high abundance of ACAGT motif in the *Trichoplax *genome represents an intriguing topic for future investigations of its potential roles in animal development and genome evolution.

## Abbreviations

TE: transposable element; LTR: long terminal repeat; ORF: open reading frame; TIR: terminal inverted repeat; EST: expressed sequence tag; MITE: miniature inverted-repeat transposable element; TSD: target site duplication; RT: reverse transcriptase; TFBS: transcription factor binding site; GO: gene ontology.

## Competing interests

The authors declare that they have no competing interests.

## Authors' contributions

SW conceived the study. SW, LZ and EM conducted bioinformatic analyses. SW, LZ, EM and ZB discussed and interpreted the results. SW, EM and ZB drafted the manuscript.

## Reviewers' comments

**Reviewer 1 **(Dr. Jerzy Jurka, Genetic Information Research Institute, USA)*This paper analyzes repetitive DNA in an interesting metazoan. Of particular interest is the predominance of the (ACAGT)n microsatellite. However, it is difficult to evaluate the analysis of TEs. The authors should include all the identified TE sequences in a supplemental file*.

**Authors' response: **Done. All identified TE sequences and annotations are included in the additional file 2.

*Furthermore, they should include analysis of non-autonomous elements if they are present. The paper needs a second review*.

**Authors' response: **We have used the MUST program to search for miniature inverted-repeat transposable elements (MITEs), which belong to nonautonomous DNA transposons, and are usually present in eukaryotic genomes in very high copy numbers. We considered a potential MITE family by requiring at least 3 elements in this family with the same perfect TIRs and TSDs. MITEs turned out to be quite rare in the genome, with only a single family represented by ~20 copies altogether. This is anticipated since no functional autonomous TEs, upon which MITEs rely for their transposition and persistence, were identified in the *Trichoplax *genome.

Reviewer 1's second review:

*Here are putative non-autonomous DNA transposons published in October issue of Repbase Reports*.

**Authors' response: **We appreciate the reviewer kindly providing this information. It is now mentioned and cited in the revised MS.

**Reviewer 2 **(Dr. I. King Jordan, Georgia Institute of Technology, USA)*This manuscript describes an analysis of the short tandem repeat and long interspersed repeat, i.e. transposable element (TE), content of the Trichoplax adhaerens genome. An analysis of the repeat content of this genome is potentially interesting because the organism has a small compact genome and it occupies a basal position in the eukaryotic phylogenetic tree. Indeed, it has been claimed previously that Trichoplax likely resembles an ancestral eukaryotic genome. As such, genomic studies of this organism may reveal insight into the origin and evolution of eukaryotic genomes. The paper reports on a straightforward analysis, and it is well written and easy to follow. However, it is not clear what truly new or relevant insight into eukaryotic genome evolution is provided by these data. In addition, the methods used to search for repeats are not sufficiently rigorous to justify the conclusions that are made regarding the repeat content of the genome. I elaborate on these points and provide more specific comments below*.

*The most pressing point here is related to the authors' contention that Trichoplax represents an ancestral eukaryotic genome and therefore its repeat content can be understood to resemble that of the earliest eukaryotes. The problem is that repeats are notoriously dynamic genomic elements. Tandem repeats are highly unstable, and TEs are typically the most lineage-specific sequences in eukaryotic genomes. In fact, TEs are known quickly evolve beyond the ability to be recognized with homology based methods. Thus, the interspersed repeats that exist in the genome today, in particular those that can be found by the methods used here, were certainly not around in at the origin of the eukaryotes. Indeed, many of the TEs identified here seem to have been recently acquired and are rapidly decaying. It does not seem possible, based on the analysis of a single genome as reported here, to determine whether the low repeat content of Tichoplax is due to low repeats in the eukaryotic ancestor or secondary loss of repeats and genome streamlining over time*.

**Authors' response: **We agree with this comment. The corresponding discussion has been removed from the MS.

*Given the small size of the Trichoplax genome, along with its basal phylogenetic position in the eukaryotic tree, it is perhaps unsurprising that the authors turn up so few TEs. However, the overall lack of TEs reported here places a burden of proof on the authors that has not been met. It is up to them to demonstrate that they have exhaustively searched the genome sequence for TEs using a wide variety of available methods. The report indicates that BLAST was used to search for encoded protein sequences and a single ab initio method was used to search for MITEs. There are of course numerous tools available to search for TEs and repeats in genome sequences (e.g. see Bergman and Quesneville 2007 Brief Bioinform 8: 382). In fact, the most rigorous efforts at genome annotation now involve pipelines that combine the use of many tools - including both homology based detection methods based on comparisons between genome sequences and TE consensus sequences and ab initio methods that rely on specific structural features of the elements (e.g. see Estill and Bennetzen 2009 Plant Methods 5: 8; Quesneville et al. 2005 PLoS Comput Biol. 1: 166). A deeper analysis of the repeat content of this genome would require such a combined approach*.

**Authors' response: **The low TE content of the *Trichoplax *genome has been first reported in the previous study [1] where 665 putative TEs were identified using the RepeatMasker program although no data curation and TE characterization were performed. In a search for nonautonomous TEs in the *Trichoplax *genome, only five putative nonautonomous DNA transposons with low copy numbers have been identified so far [14]. Besides our tblastx comparison and MITE analysis, we also performed an additional analysis to search for novel LTR retrotransposons based on their structural features. Ten putative LTR retrotransposons were identified using the LTR_FINDER program [11], and none of them showed protein homology to known LTR retrotransposons or other TEs. Moreover, none of them were present in the genome with more than two copies, so it looks unlikely that these elements are true LTR retrotransposons. Overall, we conclude that the TE content of the *Trichoplax *genome is indeed very low, and that this observation is robust across a variety of methods.

*Only a cursory description of the methods used to search for repeats are provided in the body of the manuscript. An additional description of the methods was provided by the authors upon request. These methodological details need to be included with the submission (perhaps as a supplement?) so that interested readers can more carefully evaluate the research design and the results*.

**Authors' response: **We have now provided a complete description of methods in the additional file 1.

There are several statements regarding the relevance and the impact of the findings that are never substantiated or followed up on. For instance, in the abstract the authors state that "the unexpected abundance of [the ACAGT] motif makes this an attractive target for future studies into animal development and genome evolution." And in the body of the manuscript they claim that "Identification of the new phylogenetic clade, Tag may provide new insights into the origin and diversification of the gypsy-like LTR retrotransposons in metazoan genomes." It is not clear what either of these strictly descriptive findings regarding Trichoplax genome repeats reveals about the organisms evolution or development. How does the abundance of a short tandem relate to the development of this organism? What does the discovery of a new gypsy clade, nested squarely within the diversity of existing gypsy-like sequences, tell us about the origin and diversification of the group?

**Authors' response: **1) We added more analyses to explore the potential functions of ACAGT motif. We have rewritten the discussion in the light of new results. See the following:

Many studies have shown that microsatellites can serve as transcription factor binding sites (TFBSs) to regulate gene expression [for a review, see [19]]. In order to evaluate if ACAGT motif could be a potential TFBS, we investigated the association pattern of ACAGT motif and its downstream nearby genes (Fig. 2, more info in additional file 5). Strikingly, 54% and 85% of ACAGT motif located within 1 kb and 5 kb upstream of nearby genes respectively, suggesting the potential role of ACAGT motif in regulation of nearby gene expression. Further gene ontology (GO) enrichment analysis of ACAGT associated genes (GO term level = 6 and distance threshold = 5 kb) revealed that 58 genes significantly enriched in the GO term of translation (adjusted p < 0.021), and 155 in the protein modification process (adjusted p < 0.0064). Most of the translation genes are ribosomal proteins, and most of the protein modification genes are kinases. Kinases are known to regulate the majority of cellular pathways, especially those involved in signal transduction. Previous study has shown that Trichoplax genome encodes a rich array of transcription factors and signaling pathways that are typically associated with eumetazoan developmental patterning and cell-type specification [1]. It would therefore be interesting to explore the potential roles of ACAGT motif in these biological processes in the future.

2) The phylogenetic tree of gypsy group presented in this study is an unrooted tree, and thus no ancestry information could be inferred from this tree. No outgroup was included in the phylogenetic analysis because we could not obtain a well-defined phylogeny based on the limited RT protein sequences when outgroup sequences were added. However, since most of placozoan *gypsy*-like elements belong to the *Tag *clade, and this new clade has not been identified before in other metazoan genomes, this may suggest that much of the diversity among *gypsy*-like clades emerged after the divergence of *Trichoplax *from other metazoan lineages. It is also possible that *Tag *clade may represent an ancestral *gypsy *clade which was still preserved in the *Trichoplax *genome. Further identification and characterization of full-length elements belonging to the *Tag *clade from other eukaryotic organisms would help clarify this situation, and may also provide new insights into the origin and diversification of the *gypsy*-like LTR retrotransposons.

*In summary, given the abundance of new genomes that are constantly being sequenced, one has to wonder about the need to publish a description of the repeat content, or other specific aspects of genome architecture, in each case. It would seem that to justify such an exercise, the work must provide some fundamental new insight or at the very least clearly address a specific hypothesis. This report does not meet those standards, and so I am left to wonder as to the potential impact and overall relevance of the work*.

**Authors' response: **In the revised MS, we present new analyses and discussion to expand on the previous statements. We feel that the unusual features of repeat content in *Trichoplax *are noteworthy, and that our analysis provides a useful review of those features and calls attention to a particular sequence motif that appears to be significantly associated with translation and signaling genes. We hope the reviewer will find that our MS has been improved enough to justify its publication as a Discovery Note.

Reviewer 2's second review:

*I am satisfied with the authors' responses to my comments and support publication of the revised manuscript as a Discovery Note in Biology Direct*.

## Supplementary Material

Additional file 1 Supplementary methods. Click here for file

Additional file 2 Summary of 139 putative TEs detected in the *Trichoplax *genome. Click here for file

Additional file 3 An alignment of *Ta1-11 *and other *gypsy*-like reverse transcriptase (RT) protein sequences using the ClustalW method (fasta and "aligned" formats). Click here for file

Additional file 4 Characterization of microsatellites in the genome and EST sequences of the placozoan *Trichoplax adhaerens*. Click here for file

Additional file 5 Summary of ACAGT motif and its downstream nearby genes. Click here for file

Additional file 6 The distribution of abundance and average copy number in minisatellites. Click here for file
