# Supplementary material for: Genome-wide analysis of transposable elements and tandem repeats in the compact placozoan genome
Source: Biol Direct. 2010 Apr 15;5:18. doi: 10.1186/1745-6150-5-18 (PMC2871265; doi:10.1186/1745-6150-5-18)
Supplement: Additional file 1 — Supplementary methods. [file 1745-6150-5-18-S1.PDF]

Supplementary methods

Sequences

In total, 3217 whole genome shortgun (WGS) contig sequences of *Trichoplax adhaerens* were available and downloaded from the NCBI database (GenBank accession no. ABGP01000001-ABGP01003217), which counted up to ~ 95Mb and covered 93.1% of the genome [1]. There were also 14,572 expressed sequence tags (ESTs) available for this species, which were downloaded from the JGI website (http://www.jgi.doe.gov/trichoplax).

Identification of putative transposable elements (TEs)

We adopted both homology-based and *ab initio* methods to search for putative TEs. For the homology-based method, putative TEs were identified by tblastxing *Trichoplax* contig sequences against the Repbase 14.04 database [2]. A cutoff value of $e \leq 10^{-4}$ was used as the significance threshold for the comparison. In order to check if there were any functional TEs in the *Trichoplax* genome, we also performed the tblastx comparison between *Trichoplax* ESTs and the Repbase database using the same e-value cutoff. The outputs of tblastx comparison were carefully inspected, and significant matches to simple tandem repeats and pseudogenes were excluded in the subsequent analysis. For the *ab initio* methods, long terminal repeat (LTR) retrotransposons and miniature inverted-repeat transposable elements (MITEs) were searched in the *Trichoplax* genome based on their respective structural features. Identification of LTR retrotransposons was performed using the LTR_FINDER program [3] under default parameter settings (without specifying tRNA database). MITE analysis was carried out using the MUST program [4] under default parameter settings. We considered a potential MITE family by requiring at least 3 elements in this family with the same perfect TIRs and target site duplications (TSDs).

Phylogenetic analysis of gypsy-like elements

Reverse transcriptase (RT) protein sequences were aligned using the ClustalW method [5]. The protein alignment is available in the additional file 1. Phylogenetic analysis was performed with the program MrBayes 3.1 [6]. The appropriate model of evolution was identified as WAG+G+I [7] using the MCMC model-jumping method. The MCMC chain was run for 1,000,000 generations with a sample frequency of 200. In total, 5000 trees were produced, of which the first 4500 were discarded as burn-in while summarizing the data.

Microsatellite and minisatellite mining

Microsatellite (repeat units within 1-6 bp) were detected using the SciRoko program [8]. A previous study had surveyed and analyzed the abundance and distribution of microsatellites in diverse eukaryotic taxonomic groups [9]. In order to compare our findings with their results, we adopted their searching criteria, that is, requiring perfect repeats > 12 bp.

For minisatellite mining, we used the program Tandem Repeat Finder 4.03 [10], which searched for repeat units between 7-2000 bp. The default parameter settings were used except the maximum period size parameter was set as 2000.

Associating ACAGT motif with its downstream nearby genes

Annotation information (e.g. gene name, physical location, and GO annotation) of 11,520 *Trichoplax* genes was obtained from the JGI website (http://www.jgi.doe.gov/trichoplax). Because these information was generated based on the masked scaffold sequences, we recalculated the frequency of ACAGT motif in these masked scaffold sequences, that is, 5548. An associated gene is determined if: (i) ACAGT motif is located at upstream of a given gene, and (ii) the distance between ACAGT motif and this gene is minimal.

To test whether the ACAGT motif was associated with a particular functional category of genes, or was randomly distributed among all genes, we selected the set of 4,719 genes most closely

associated with this motif (≤5 kb). For those genes annotated with Gene Ontology (GO) terms, we first standardized all terms to the same level (Gene Ontology level 6), based on the ontology files downloaded from [11]. We then counted the number of genes associated with each process at that level. We compared these data with the number of genes in the complete genome associated with each of those processes, using Fisher's Exact Test, corrected for multiple tests using the methods presented in [12]. These comparisons revealed that this motif was more frequently encountered in association with genes from two biological processes (GO:0006412, translation; and GO:0006464, protein modification) than would be expected by chance.

References

1. Srivastava M, Begovic E, Chapman J, Putnam NH, Hellsten U, Kawashima T, Kuo A, Mitros T, Salamov A, Carpenter ML, Signorovitch AY, Moreno MA, Kamm K, Grimwood J, Schmutz J, Shapiro H, Grigoriev IV, Buss LW, Schierwater B, Dellaporta SL, Rokhsar DS: **The *Trichoplax* genome and the nature of placozoans.** Nature 2008, **454:** 955-960.

2. Jurka J, Kapitonov VV, Pavlicek A, Klonowski P, Kohany O, Walichiewicz J: **Repbase Update, a database of eukaryotic repetitive elements.** Cytogenet Genome Res 2005, **110:** 462-467.

3. Xu Z, Wang H: **LTR_FINDER: an efficient tool for the prediction of full-length LTR retrotransposons.** Nucleic Acids Res 2007, **35:** W265-W268.

4. Chen Y, Zhou F, Li G, Xu Y: **MUST: A system for identification of miniature inverted-repeat transposable elements and applications to *Anabaena variabilis* and *Haloquadratum walsbyi*.** Gene 2009, **436:** 1-7.

5. Bouneau L, Fischer C, Ozouf-Costaz C, Froschauer A, Jaillon O, Coutanceau J-P, Korting C, Weissenbach J, Bernot A, Volff J-N: **An active non-LTR retrotransposon with tandem structure in the compact genome of the pufferfish *Tetraodon nigroviridis*.** Genome Res 2003, **13:** 1686-1695.

6. Jaillon O, et al.: **Genome duplication in the teleost fish *Tetraodon nigroviridis* reveals the early vertebrate proto-karyotype.** Nature 2004, **431:** 946-957.

7. Thompson JD, Higgins DG, Gibson TJ: **CLUSTAL W: improving the sensitivity of progressive multiple sequence alignment through sequence weighting, position-specific gap penalties and weigh matrix choice.** Nucleic Acids Res 1994, **22:** 4673-4680.

8. Malik H, Eickbush TH: **Modular evolution of the integrase domain in the *Ty3/Gypsy* class of LTR retrotransposons.** J Virol 1999, **73:** 5186-5190.

9. Bae Y-A, Moon S-Y, Kong Y, Cho S-Y, Rhyu M-G: ***CsRn1*, a novel active retrotransposon in a parasitic trematode, *Clonorchis sinensis*, discloses a new phylogenetic clade of *Ty3*/*gypsy*-like LTR retrotransposons.** Mol Biol Evol 2001, **18:** 1474-1483.

10. Benson G: **Tandem repeats finder: a program to analyze DNA sequences.** Nucleic Acids Res 1999, **27:** 573-580.

11. http://www.geneontology.org/GO.downloads.shtml

12. Benjamini Y, Hochberg Y: **Controlling the false discovery rate: a practical and powerful approach to multiple testing**. J R STAT SOC B 1995, **57:** 289–300.
