# Supplementary material for: Genome-wide analysis of transposable elements and tandem repeats in the compact placozoan genome
Source: Biol Direct. 2010 Apr 15;5:18. doi: 10.1186/1745-6150-5-18 (PMC2871265; doi:10.1186/1745-6150-5-18)
Supplement: Additional file 3 — An alignment of Ta1-11 and other gypsy-like reverse transcriptase (RT) protein sequences using the ClustalW method (fasta and "aligned" formats). [file 1745-6150-5-18-S3.PDF]

A. An alignment of *Ta1-11* and other *gypsy*-like reverse transcriptase (RT) protein sequences using the ClustalW method (fasta format)

```
>Ta1
---------------------------------------------------------------------------------------------------------------FA-
MFS---MPFGLHSAP-ATFQRLMNAVL-KDLQG-VANV-LDDTIVYSASWKEHLQHLGTGIRSIATIRIKC-RIKVQFRKQ----------------------
>Ta2
--------------------------------------------------------------------------------------------------------------LFA-
MFS---MPFGLHCAP-ATFQRLMNAVL-KDLQG-VASVYLDDTIVYSANWKEHLQHLGTGIRSIATIWIKCYRRKAQFRKQ----------------------
>Ta3
---------------SSPM-LVKK---------------PYL---SYRVCIDFRMINPLSI-DA-YPFP-LDEIL-LLRRPRYISTLELAKGY-QIPLGNRS-AITGFATSLW-
LFKFRVMPFDMHSAP-ATFQR----------------------------------------------------------------------------------
>Ta4
------------SPWASPLMLVRKKN------------------GTYRPCVDFRRVNAVTEMDA-YPIPKITEILDRIRQAKFISTIDQSKGYGQIPLPPNSQKTTAFITPFG-
LYEFKVMPIGLHSAP-ATFQRLMDAVL-KDLPG-VASVYLDDIMFYSANWKEHLQHLELVFDR-RQYGFSVKEEKCNFACNIVSYLG-HTI------------
>Ta5
--------------------------------------------------------------------------------------LDLSKGYWQIPIEEESKSYTDFTTPIG-
LYEFNTMPFGLQNAP-VTFKSLVNSLF-AEYSE-YVAVCLDGIVIFDQTWEQHLSHIANVFKILEAAKLTL-PSKCIFAEGTVNYLG-HQIG--SGKLKP---
>Ta6
------------SPWASPVVLVKKPN------------------GTFRPCIDYRKLNSCTKADA-YPLPRIGYILDIVGNAKFITMLDLSKGYCQIPMEEKSKSYTAFTTPFG-
LIEFNVMPFGLHNAP-GTFQQLVNTLF-GKHYE-YLAVYMDDIIIFDTSWEDHSKHLEIIFKVLEDANLKLQPSKCTFAKGTVTYLG-HQIG--SGLVKP---
>Ta7
------------SPWASPVILVKKPN------------------GTYRPCVDYRKLNACTTVNA-YPLPRIDDLLDTVGNAKFITTLDLSKGYWQIPMEEGSKQYTAFTTPYG-
LYEFEMMPFGLHNAP-ATFQSLVNSLF-AHISD-FVAVYLDDIVV----------------------------------------------------------
>Ta8
------------SPWASPVILVKKPN------------------GTYRPCVDYRKLNACTTVNA-YPLPRIDDLLDIVGNAKFITTLDLSKGYWQIPMEEGSKQYTAFTTPYG-
LYEFEMMPFGLHNAP-ATFQSLVNSLF-AHISD-FVAVYLDDIVVYDTTWEDHLRHLKTVFQILEAANLTLQPAKCTFAKGTVTYLG-HQIG--SGVVQP---
>Ta9
--------------------------------------------------------------------------------------------------------------------
-------GIASAP-AIFQQLMDTMLQGIPGT---ACYLDDIIVTGKTKAEHPQSLKQVLDGINTSGFSVQLNKCSFMQGKIKYLG-HVLDKNGIHMSQKKV
>Ta10
---GILESI-EHYEWATPIVAIPKAL------------------DAVRICGDFKVTVNPVLCTDQYPLPRSEELFAALNGGSLFTKLDFSEAYLQVELDPASRKYLVINTHKG-
LYRYTRLPFGIASAPPTIFQQLMDTMLQGIPGT---VCYLDDIIVTGKTTQEHLQSLQQVLDRIHTYGFRVKLNKCSFMQKRIEYLG-HVIDKDGVHMSPRKV
>Ta11
------------SPYAAPVVLVPK---------------PDG---KWRFCVDYRGLNKVSIRDV-YPLPRIDDSLDVLGHAKYFSTLDFFSGYWQVPVAEEDKAKTAFITHNG-
LYEFNVMPFGLTNAP-ATFQRLMNLLLTGLCWI-NCIVYIDDVIVYSNTFNMHLQHLEEVFKRVDRANLKLKLAKCIFAAREVKFLG-HIISADGILPNPDKI
>Jule
EKMKVISKV-EKSEWASPIVTVPKTD------------------KTIRICGDYKVSINQCIEEKHYPLPNAEDLFATLEGGTSFSKLDLSHAYQQLELDEESEKYLVINTHKG-
LYKYSRLSYGVSSAP-AIFQSVMDQILQGMDHV---TCFLDDILITASSEKEHLQRLEEVLTRLEKHGLRVKLAKCRFLQSSVEYLG-HRIDKDGLHPMEEKV
```

>Mag
LAAGVIKPV-DHSDWATPLVVVRKAD------------------GGLRICADYKVTLNKVLAIDRFPVPKMEDLFSNLSGNKFFTKLDLSQAYNQIVLSERSSEYTVINTHRG-
LFKYSRLVYGLASSP-GIFQKLMVNMFKNVPNV---VVFYDDILIRNQDLDSHLKSIKEVLDILERYGLKIKRSKCEFMVTEVRYLG-FIIDQNGVRVDPEKV
>SURL
ERLDVITPVDEPTDWVSSLVVVMKKN------------------GQLRVCLDPRD-LNRAIKREHYQLPSRAEITAHFAGAKYFSKLDASSGFWQIQLDDESSKLCTFITPYG-
RYKFLRLPFGICSAP-EVYHKIVHQMFAHIPGV---NTMMDDVIVWGTTQQEHDNRLREVLSIARRMNLKLNKDKCEFSVKKLTFIG-DLISDQGVQPDPKKV
>Gulliver
-YMNVISPV-SYS-VATPLVFIKKAD------------------GKLRVCGDFSTGLNKALQDHLYPLPIPEDLFTILNGCVVFSKIDFSDAYFQVEVAEESRLLLTVNTHRG-
LFQYNRLPFGVKCAP-AIFQQIMDTMLADLPFS---MAYMDDIIVTSKNEKEHYAHSQKVFRRLSEYGFCMKLEKCSFFMSSIKYLG-IIIDLNGGRPNPEKV
>Fugitive
QQAGVIQTG-NYSAWAAPIVVIRKPN------------------GTIRICADFSTGLNAALDEYQYPLPVPEDLFAKLNGGTCFAKIDFSDAYLQVEVDDDSRGLLTINTHRG-
LFQYTRLPFGIKTAP-AIFQQIMDTMLTGIPGA---AAYLDDIIVMGSTNSELSDRLHQVLNRVLEYGFQLREEKCIFFMHSIKYLG-FILDKNGRRPDPANI
>Cer1
-LNQKVIRESK-SPWSSPVVLVKK---------------KDG---SIRMCIDYRKVNKVVKNNA-HPLPNIEATLQSLAGKKLYTVFDMIAGFWQIPLDEKSKEITAFAIGSE-
LFEWNVLPFGLVISP-ALFQGTMEEIIGDLLGV-CAFVYVDDLLIASKDMEQHLQDVKEALTRIRKSGMKLRASKCHIAKKEVEYLG-HKVTLDGVETQEVKT
>CsRn1
-LELGIIRTSS-SHWSSPLHMVPKK-----------------SKGDWRPCGDYRSLNYATIPDR-YPIPHIHDFASTLCHTNIFSKLDLVRAYYHIPVAPDDIPKTAITTPFG-
LFEFTRIPFGLRNAA-QTFQRFMDEVLRGLP---FVYAYLDDVLIASTSPTEHAAHLRAVFERLSTYSIRLNIDKCLFGVTSLDFLG-HHIDSTGISPLPDRI
>Gypsy
-LKDGIIRPSR-SPYNSPTWVVDKKGT------------DAFGNPNKRLVIDFRKLNEKTIPDR-YPMPSIPMILANLGKAKFFTTLDLKSGYHQIYLAEHDREKTSFSVNGG-
KYEFCRLPFGLRNAS-SIFQRALDDVLREQIGK-ICYVYVDDVIIFSENESDHVRHIDTVLKCLIDANMRVSQEKTRFFKESVEYLG-FIVSKDGTKSDPEKV
>Mdg1
-IKDGIVEQSI-SEYNSPLLLVPKKSL------------PNSEEKRWRLVVDYRQINKKLLADK-FPLPRIEDILDQLGRAKYFSCLDLMSGFHQIELDERSRNITSFSTSTG-
AYRYTRLPFGLKIAP-NSFQRMMTLAFSGLTPS-QAFLYMDDLVVIGCSEKHMLKNLTDVFKLCRQHNLKLHPEKCTFFMKEVTYLG-HKCTDKGILPDDSKY
>Mdg3
-LENGFIRPSD-SEYASPIVLVKKK------------------TGDLRMCVDFRKLNKMTMKDN-YPLPLIDDLLDRMNEKTVFTKLDLKNGFFHVHVKKESIKYTSFVTPLG-
QYEWLRMPFGLKNAP-SVFQRFVNKIFADMIRENKVVVYMDDILLATENINEHLETLKEIFKRLVENKLELRIDKCEFMQSSIKYLG-FIINKDGIMPNDKGI
>Osvaldo
--LLGVEYRAR-KSTEEWTVGGKVVR------------------GGFQWRKRSRNATGSDPGGS-
YPLPRVHHILDQLREARYITSLDLKDGYWQIPMEKSSRPLTAFTVPGKGLLQWKVMPFGLHSAP-VTFQRALDQVIGPDMMP-
HAFAYLDDKIVIGRTRQEHMDNLREMFRRLRAANLRINIYKCDFFKKELIYLG-HKVTEDGIRTDPEKV
>Cyclops
-LDARMIYPISDSPWVSPVHVVPKKGGNTVIRNDKDELIPTKVATGWRMCIEYRRLNTATRKDH-FPLPFMDQMLERLSGQQYYCFLDGYSGYNQIAVDPADHXKTAFTCPFG-
VFAYRKMSFGLCNAP-TTFQRCVQAIFADLNEK-TMEVFMDDFSVFGVSFSLCLANLKTVLERCVKTNLVLNWXKCHFMVTEGIVLG-HKVSSRGLEVDRAKV
>Blastopia
-VEQSIVRKST-SNVASRIVVVRKK------------------DGTLRVCVDYRKLNTMVLMDC-FPVPIMEEVLEKLQSAKWFTTMDLQNGFFHVAVEEASKPYTAFVTREG-
LFEFNKAPFGFKNSP-AAFIRFVQFIFQELINSNIMQLYMDDIIVYAATPEECMEKTEMVLKRAAEFGLKIKWKKCNFMQRRIHFLG-HIIEGGQICPGKEKT
>Athila

-LDAGIIYPISDSTWVSPVHVVPKKGGVTVVKNDNDELIPTRTITGHRMCIDYRKLNAASRKDH-FPLPFIDQMLERLANHTHYCFLDGYSGFFQIPIHPNDQEKTTFTCPYG-
TFAYRRMPFGLCNAP-ATFQRCMMSIFSDLIEN-VVEVFMDDFSVYGDSFASCLSNLCRVLKRCEETNLVLNWEKCHCMVRDGIVLG-HKISEKGIEVDRAKI
>Woot
-LDLGVIKREA-SPYASPMTVGKKKD------------------GTVRICLDARMINSKMIADC-ESPPAADELLRRFHEIRYMSTIDLRSSYWQIPLSPESRQYTAFLYNGR-
SYTYQVLPFGLKTAV-GSFSRAMDVVLGTEVRE-FVVNYIDDLLVASETLNEHLEHLRQVFEKLKQARMTINLEKSNFIQKE-KFLG-HILTINGIKADPEKI
>Ulysses
-LKLGIIEESD-SPWSNRTTVVMRP-------------------GKNRFCLDARKLNSVTVKDA-YPLPCIEGILRDRRDSHFISSVDLKFAFWQIEMEEKSRAYTAFTVPRP-
LYQFRHMPFGLCNAA-QHFERLMDKVIPANLRS-NVFVYLDDLLIISADFPTHLKYLELVAECLRNANLTIGMAKSKFLFRNLNYLGFIQLRRRTWRMDPGRV
>Tom
-LEQGLIRESN-SPYNSPTWVVPKK-P------------DASGKAKYRVVIDYRKLNEITIPDR-FPIPNMDEILGKLGKCQYFTTIDLARGFHQIEMDSESIQKTAFSTKRG-
HYEYVRMPFGLRNAP-ATFQRCMNNILRPLINK-HCLVYLDDMIIFSTSLDEHLNSLQLVFEKLSESNLKLQLDKCEFLKKEATFLG-HIVTPDGIKPNPLKV
>TED
-LDQGIIRPSD-SAWSSPIWVVPKK-I------------DASGKQKWRLVVDFRKLNEKTIDDK-YPIPNISDVLDKLGKCQYFTTLDLASGFYQVEMDPQDISKTAFNVEHG-
HFEFLRMPMGLKNSP-STFQRVMDNVLRGLQNN-ICLVYLDDIIVYSTSLQEHLENLERVFQRLRESNFKIQMDKSEFLKLETAYLG-HIISRDGIKPNPDKI
>Yoyo
-LEDGIIRPSR-SPYNSPVWIVDKK-P------------DSLGNKQYRLVIDYRKLNSVTIADR-YPIPEINEVLSHLGSNTFFSVIDLKSGFHQIPLKNSDIEKTAFSINNE-
KYEFTRLPFGLKNAP-SIFQRTLDDILRDYIGQ-CCYVYIDDIIIFSRNEKEHSTHLKNIFTTLEKANMKVQLDKCKFFEKEVEFLG-FIVTPEGIKTNPSKI
>412
-IKDKIVEPSV-SQYNSPLLLVPKKSS------------PNSDKKKWRLVIDYRQINKKLLADK-FPLPRIDDILDQLGRAKYFSCLDLMSGFHQIELDEGSRDITSFSTSNG-
SYRFTRLPFGLKIAP-NSFQRMMTIAFSGIEPS-QAFLYMDDLIVIGCSEKHMLKNLTEVFGKCREYNLKLHPEKCSFFMHEVTFLG-HKCTDKGILPDDKKY
>Sushi
-LASGIIRPSS-SPLAAGFFFVAKE------------------DGGLRPCIDFRKLNNITVKNK-YPLPLMSSTFEPLTHARVFTKLDLRNAYHLVQIRKGDEWKTAFNTHLG-
HFEYLVMPFGLSNAP-AVFQELVNDVLRDMINV-FVVVYLDDILIFSRTMEEHHQHVRLVLQRLLENRLFIKAEKCIFHSASVGYLG-YIVEEGRVRADPAKI
>Del
-LNKGFIRGST-SPWGAHVLFDPKK------------------DDSKRMCIDY-KLNSVTVKNK-YPLPRIDDLFDQLNGA-YFSKIDLRFRYHQLRIR-ADIPKTAFRTRYG-
HYEFLVMPFGLTNVP-TAFMNLMNRVFREYLDK-FIVVFVDYVLIYSRTQKDHEHHLRISLQLLRNNQLYAKLSKCEFWMEKVKFLG-HVVSREGIVVDPVKV
>Maggy
-LKKGFIRPSS-SSVASPVLFVKKQ------------------GGGLRFCVDYRALNNITVKDR-YPLPLVRETLNNLAGMKFFSKIDIVSAFNNIRIKKGEEYLTAFRTRFG-
LYESLVMPFGLTGAP-ATFQRYINDSLREYLDV-FCTAYLDDILIYSRTRTEHEEHLKLVLEALRKAGLYANAAKCEFFVTETKFLG-LLVGVEGVKMDPEKI

B. An alignment of *Ta1-11* and other *gypsy*-like reverse transcriptase (RT) protein sequences using the ClustalW method ("aligned" format)

```
Ta1             ------------------------------------------------------------
Ta2             ------------------------------------------------------------
Ta3             ---------------SSPM-LVKK---------------PYL---SYRVCIDFRMINPLS 26
Ta4             ------------SPWASPLMLVRKKN------------------GTYRPCVDFRRVNAVT 30
Ta5             ------------------------------------------------------------
Ta6             ------------SPWASPVVLVKKPN------------------GTFRPCIDYRKLNSCT 30
Ta7             ------------SPWASPVILVKKPN------------------GTYRPCVDYRKLNACT 30
Ta8             ------------SPWASPVILVKKPN------------------GTYRPCVDYRKLNACT 30
Ta9             ------------------------------------------------------------
Ta10            ---GILESI-EHYEWATPIVAIPKAL------------------DAVRICGDFKVTVNPV 38
Ta11            ------------SPYAAPVVLVPK---------------PDG---KWRFCVDYRGLNKVS 30
Jule            EKMKVISKV-EKSEWASPIVTVPKTD------------------KTIRICGDYKVSINQC 41
Mag             LAAGVIKPV-DHSDWATPLVVVRKAD------------------GGLRICADYKVTLNKV 41
SURL            ERLDVITPVDEPTDWVSSLVVVMKKN------------------GQLRVCLDPRD-LNRA 41
Gulliver        -YMNVISPV-SYS-VATPLVFIKKAD------------------GKLRVCGDFSTGLNKA 39
Fugitive        QQAGVIQTG-NYSAWAAPIVVIRKPN------------------GTIRICADFSTGLNAA 41
Cer1            -LNQKVIRESK-SPWSSPVVLVKK---------------KDG---SIRMCIDYRKVNKVV 40
CsRn1           -LELGIIRTSS-SHWSSPLHMVPKK-----------------SKGDWRPCGDYRSLNYAT 41
Gypsy           -LKDGIIRPSR-SPYNSPTWVVDKKGT------------DAFGNPNKRLVIDFRKLNEKT 46
Mdg1            -IKDGIVEQSI-SEYNSPLLLVPKKSL------------PNSEEKRWRLVVDYRQINKKL 46
Mdg3            -LENGFIRPSD-SEYASPIVLVKKK------------------TGDLRMCVDFRKLNKMT 40
Osvaldo         --LLGVEYRAR-KSTEEWTVGGKVVR------------------GGFQWRKRSRNATGSD 39
Cyclops         -LDARMIYPISDSPWVSPVHVVPKKGGNTVIRNDKDELIPTKVATGWRMCIEYRRLNTAT 59
Blastopia       -VEQSIVRKST-SNVASRIVVVRKK------------------DGTLRVCVDYRKLNTMV 40
Athila          -LDAGIIYPISDSTWVSPVHVVPKKGGVTVVKNDNDELIPTRTITGHRMCIDYRKLNAAS 59
Woot            -LDLGVIKREA-SPYASPMTVGKKKD------------------GTVRICLDARMINSKM 40
Ulysses         -LKLGIIEESD-SPWSNRTTVVMRP-------------------GKNRFCLDARKLNSVT 39
Tom             -LEQGLIRESN-SPYNSPTWVVPKK-P------------DASGKAKYRVVIDYRKLNEIT 45
TED             -LDQGIIRPSD-SAWSSPIWVVPKK-I------------DASGKQKWRLVVDFRKLNEKT 45
Yoyo            -LEDGIIRPSR-SPYNSPVWIVDKK-P------------DSLGNKQYRLVIDYRKLNSVT 45
412             -IKDKIVEPSV-SQYNSPLLLVPKKSS------------PNSDKKKWRLVIDYRQINKKL 46
Sushi           -LASGIIRPSS-SPLAAGFFFVAKE------------------DGGLRPCIDFRKLNNIT 40
Del             -LNKGFIRGST-SPWGAHVLFDPKK------------------DDSKRMCIDY-KLNSVT 39
Maggy           -LKKGFIRPSS-SSVASPVLFVKKQ------------------GGGLRFCVDYRALNNIT 40
```

```
Ta1        ---------------------------------------------------FA-MFS--- 5
Ta2        --------------------------------------------------LFA-MFS--- 6
Ta3        I-DA-YPFP-LDEIL-LLRRPRYISTLELAKGY-QIPLGNRS-AITGFATSLW-LFKFRV 79
Ta4        EMDA-YPIPKITEILDRIRQAKFISTIDQSKGYGQIPLPPNSQKTTAFITPFG-LYEFKV 88
Ta5        --------------------------LDLSKGYWQIPIEEESKSYTDFTTPIG-LYEFNT 33
Ta6        KADA-YPLPRIGYILDIVGNAKFITMLDLSKGYCQIPMEEKSKSYTAFTTPFG-LIEFNV 88
Ta7        TVNA-YPLPRIDDLLDTVGNAKFITTLDLSKGYWQIPMEEGSKQYTAFTTPYG-LYEFEM 88
Ta8        TVNA-YPLPRIDDLLDIVGNAKFITTLDLSKGYWQIPMEEGSKQYTAFTTPYG-LYEFEM 88
Ta9        ------------------------------------------------------------
Ta10       LCTDQYPLPRSEELFAALNGGSLFTKLDFSEAYLQVELDPASRKYLVINTHKG-LYRYTR 97
Ta11       IRDV-YPLPRIDDSLDVLGHAKYFSTLDFFSGYWQVPVAEEDKAKTAFITHNG-LYEFNV 88
Jule       IEEKHYPLPNAEDLFATLEGGTSFSKLDLSHAYQQLELDEESEKYLVINTHKG-LYKYSR 100
Mag        LAIDRFPVPKMEDLFSNLSGNKFFTKLDLSQAYNQIVLSERSSEYTVINTHRG-LFKYSR 100
SURL       IKREHYQLPSRAEITAHFAGAKYFSKLDASSGFWQIQLDDESSKLCTFITPYG-RYKFLR 100
Gulliver   LQDHLYPLPIPEDLFTILNGCVVFSKIDFSDAYFQVEVAEESRLLLTVNTHRG-LFQYNR 98
Fugitive   LDEYQYPLPVPEDLFAKLNGGTCFAKIDFSDAYLQVEVDDDSRGLLTINTHRG-LFQYTR 100
Cer1       KNNA-HPLPNIEATLQSLAGKKLYTVFDMIAGFWQIPLDEKSKEITAFAIGSE-LFEWNV 98
CsRn1      IPDR-YPIPHIHDFASTLCHTNIFSKLDLVRAYYHIPVAPDDIPKTAITTPFG-LFEFTR 99
Gypsy      IPDR-YPMPSIPMILANLGKAKFFTTLDLKSGYHQIYLAEHDREKTSFSVNGG-KYEFCR 104
Mdg1       LADK-FPLPRIEDILDQLGRAKYFSCLDLMSGFHQIELDERSRNITSFSTSTG-AYRYTR 104
Mdg3       MKDN-YPLPLIDDLLDRMNEKTVFTKLDLKNGFFHVHVKKESIKYTSFVTPLG-QYEWLR 98
Osvaldo    PGGS-YPLPRVHHILDQLREARYITSLDLKDGYWQIPMEKSSRPLTAFTVPGKGLLQWKV 98
Cyclops    RKDH-FPLPFMDQMLERLSGQQYYCFLDGYSGYNQIAVDPADHXKTAFTCPFG-VFAYRK 117
Blastopia  LMDC-FPVPIMEEVLEKLQSAKWFTTMDLQNGFFHVAVEEASKPYTAFVTREG-LFEFNK 98
Athila     RKDH-FPLPFIDQMLERLANHTHYCFLDGYSGFFQIPIHPNDQEKTTFTCPYG-TFAYRR 117
Woot       IADC-ESPPAADELLRRFHEIRYMSTIDLRSSYWQIPLSPESRQYTAFLYNGR-SYTYQV 98
Ulysses    VKDA-YPLPCIEGILRDRRDSHFISSVDLKFAFWQIEMEEKSRAYTAFTVPRP-LYQFRH 97
Tom        IPDR-FPIPNMDEILGKLGKCQYFTTIDLARGFHQIEMDSESIQKTAFSTKRG-HYEYVR 103
TED        IDDK-YPIPNISDVLDKLGKCQYFTTLDLASGFYQVEMDPQDISKTAFNVEHG-HFEFLR 103
Yoyo       IADR-YPIPEINEVLSHLGSNTFFSVIDLKSGFHQIPLKNSDIEKTAFSINNE-KYEFTR 103
412        LADK-FPLPRIDDILDQLGRAKYFSCLDLMSGFHQIELDEGSRDITSFSTSNG-SYRFTR 104
Sushi      VKNK-YPLPLMSSTFEPLTHARVFTKLDLRNAYHLVQIRKGDEWKTAFNTHLG-HFEYLV 98
Del        VKNK-YPLPRIDDLFDQLNGA-YFSKIDLRFRYHQLRIR-ADIPKTAFRTRYG-HYEFLV 95
Maggy      VKDR-YPLPLVRETLNNLAGMKFFSKIDIVSAFNNIRIKKGEEYLTAFRTRFG-LYESLV 98
```

```
Ta1               MPFGLHSAP-ATFQRLMNAVL-KDLQG-VANV-LDDTIVYSASWKEHLQHLGTGIRSIAT 61
Ta2               MPFGLHCAP-ATFQRLMNAVL-KDLQG-VASVYLDDTIVYSANWKEHLQHLGTGIRSIAT 63
Ta3               MPFDMHSAP-ATFQR--------------------------------------------- 93
Ta4               MPIGLHSAP-ATFQRLMDAVL-KDLPG-VASVYLDDIMFYSANWKEHLQHLELVFDR-RQ 144
Ta5               MPFGLQNAP-VTFKSLVNSLF-AEYSE-YVAVCLDGIVIFDQTWEQHLSHIANVFKILEA 90
Ta6               MPFGLHNAP-GTFQQLVNTLF-GKHYE-YLAVYMDDIIIFDTSWEDHSKHLEIIFKVLED 145
Ta7               MPFGLHNAP-ATFQSLVNSLF-AHISD-FVAVYLDDIVV--------------------- 124
Ta8               MPFGLHNAP-ATFQSLVNSLF-AHISD-FVAVYLDDIVVYDTTWEDHLRHLKTVFQILEA 145
Ta9               ---GIASAP-AIFQQLMDTMLQGIPGT---ACYLDDIIVTGKTKAEHPQSLKQVLDGINT 53
Ta10              LPFGIASAPPTIFQQLMDTMLQGIPGT---VCYLDDIIVTGKTTQEHLQSLQQVLDRIHT 154
Ta11              MPFGLTNAP-ATFQRLMNLLLTGLCWI-NCIVYIDDVIVYSNTFNMHLQHLEEVFKRVDR 146
Jule              LSYGVSSAP-AIFQSVMDQILQGMDHV---TCFLDDILITASSEKEHLQRLEEVLTRLEK 156
Mag               LVYGLASSP-GIFQKLMVNMFKNVPNV---VVFYDDILIRNQDLDSHLKSIKEVLDILER 156
SURL              LPFGICSAP-EVYHKIVHQMFAHIPGV---NTMMDDVIVWGTTQQEHDNRLREVLSIARR 156
Gulliver          LPFGVKCAP-AIFQQIMDTMLADLPFS---MAYMDDIIVTSKNEKEHYAHSQKVFRRLSE 154
Fugitive          LPFGIKTAP-AIFQQIMDTMLTGIPGA---AAYLDDIIVMGSTNSELSDRLHQVLNRVLE 156
Cer1              LPFGLVISP-ALFQGTMEEIIGDLLGV-CAFVYVDDLLIASKDMEQHLQDVKEALTRIRK 156
CsRn1             IPFGLRNAA-QTFQRFMDEVLRGLP---FVYAYLDDVLIASTSPTEHAAHLRAVFERLST 155
Gypsy             LPFGLRNAS-SIFQRALDDVLREQIGK-ICYVYVDDVIIFSENESDHVRHIDTVLKCLID 162
Mdg1              LPFGLKIAP-NSFQRMMTLAFSGLTPS-QAFLYMDDLVVIGCSEKHMLKNLTDVFKLCRQ 162
Mdg3              MPFGLKNAP-SVFQRFVNKIFADMIRENKVVVYMDDILLATENINEHLETLKEIFKRLVE 157
Osvaldo           MPFGLHSAP-VTFQRALDQVIGPDMMP-HAFAYLDDKIVIGRTRQEHMDNLREMFRRLRA 156
Cyclops           MSFGLCNAP-TTFQRCVQAIFADLNEK-TMEVFMDDFSVFGVSFSLCLANLKTVLERCVK 175
Blastopia         APFGFKNSP-AAFIRFVQFIFQELINSNIMQLYMDDIIVYAATPEECMEKTEMVLKRAAE 157
Athila            MPFGLCNAP-ATFQRCMMSIFSDLIEN-VVEVFMDDFSVYGDSFASCLSNLCRVLKRCEE 175
Woot              LPFGLKTAV-GSFSRAMDVVLGTEVRE-FVVNYIDDLLVASETLNEHLEHLRQVFEKLKQ 156
Ulysses           MPFGLCNAA-QHFERLMDKVIPANLRS-NVFVYLDDLLIISADFPTHLKYLELVAECLRN 155
Tom               MPFGLRNAP-ATFQRCMNNILRPLINK-HCLVYLDDMIIFSTSLDEHLNSLQLVFEKLSE 161
TED               MPMGLKNSP-STFQRVMDNVLRGLQNN-ICLVYLDDIIVYSTSLQEHLENLERVFQRLRE 161
Yoyo              LPFGLKNAP-SIFQRTLDDILRDYIGQ-CCYVYIDDIIIFSRNEKEHSTHLKNIFTTLEK 161
412               LPFGLKIAP-NSFQRMMTIAFSGIEPS-QAFLYMDDLIVIGCSEKHMLKNLTEVFGKCRE 162
Sushi             MPFGLSNAP-AVFQELVNDVLRDMINV-FVVVYLDDILIFSRTMEEHHQHVRLVLQRLLE 156
Del               MPFGLTNVP-TAFMNLMNRVFREYLDK-FIVVFVDYVLIYSRTQKDHEHHLRISLQLLRN 153
Maggy             MPFGLTGAP-ATFQRYINDSLREYLDV-FCTAYLDDILIYSRTRTEHEEHLKLVLEALRK 156
                     ..       :
```

```
Ta1             IRIKC-RIKVQFRKQ---------------------- 75
Ta2             IWIKCYRRKAQFRKQ---------------------- 78
Ta3             -------------------------------------
Ta4             YGFSVKEEKCNFACNIVSYLG-HTI------------ 168
Ta5             AKLTL-PSKCIFAEGTVNYLG-HQIG--SGKLKP--- 120
Ta6             ANLKLQPSKCTFAKGTVTYLG-HQIG--SGLVKP--- 176
Ta7             -------------------------------------
Ta8             ANLTLQPAKCTFAKGTVTYLG-HQIG--SGVVQP--- 176
Ta9             SGFSVQLNKCSFMQGKIKYLG-HVLDKNGIHMSQKKV 89
Ta10            YGFRVKLNKCSFMQKRIEYLG-HVIDKDGVHMSPRKV 190
Ta11            ANLKLKLAKCIFAAREVKFLG-HIISADGILPNPDKI 182
Jule            HGLRVKLAKCRFLQSSVEYLG-HRIDKDGLHPMEEKV 192
Mag             YGLKIKRSKCEFMVTEVRYLG-FIIDQNGVRVDPEKV 192
SURL            MNLKLNKDKCEFSVKKLTFIG-DLISDQGVQPDPKKV 192
Gulliver        YGFCMKLEKCSFFMSSIKYLG-IIIDLNGGRPNPEKV 190
Fugitive        YGFQLREEKCIFFMHSIKYLG-FILDKNGRRPDPANI 192
Cer1            SGMKLRASKCHIAKKEVEYLG-HKVTLDGVETQEVKT 192
CsRn1           YSIRLNIDKCLFGVTSLDFLG-HHIDSTGISPLPDRI 191
Gypsy           ANMRVSQEKTRFFKESVEYLG-FIVSKDGTKSDPEKV 198
Mdg1            HNLKLHPEKCTFFMKEVTYLG-HKCTDKGILPDDSKY 198
Mdg3            NKLELRIDKCEFMQSSIKYLG-FIINKDGIMPNDKGI 193
Osvaldo         ANLRINIYKCDFFKKELIYLG-HKVTEDGIRTDPEKV 192
Cyclops         TNLVLNWXKCHFMVTEGIVLG-HKVSSRGLEVDRAKV 211
Blastopia       FGLKIKWKKCNFMQRRIHFLG-HIIEGGQICPGKEKT 193
Athila          TNLVLNWEKCHCMVRDGIVLG-HKISEKGIEVDRAKI 211
Woot            ARMTINLEKSNFIQKE-KFLG-HILTINGIKADPEKI 191
Ulysses         ANLTIGMAKSKFLFRNLNYLGFIQLRRRTWRMDPGRV 192
Tom             SNLKLQLDKCEFLKKEATFLG-HIVTPDGIKPNPLKV 197
TED             SNFKIQMDKSEFLKLETAYLG-HIISRDGIKPNPDKI 197
Yoyo            ANMKVQLDKCKFFEKEVEFLG-FIVTPEGIKTNPSKI 197
412             YNLKLHPEKCSFFMHEVTFLG-HKCTDKGILPDDKKY 198
Sushi           NRLFIKAEKCIFHSASVGYLG-YIVEEGRVRADPAKI 192
Del             NQLYAKLSKCEFWMEKVKFLG-HVVSREGIVVDPVKV 189
Maggy           AGLYANAAKCEFFVTETKFLG-LLVGVEGVKMDPEKI 192
```
