# Supplementary material for: Genome-wide analysis of transposable elements and tandem repeats in the compact placozoan genome
Source: Biol Direct. 2010 Apr 15;5:18. doi: 10.1186/1745-6150-5-18 (PMC2871265; doi:10.1186/1745-6150-5-18)
Supplement: Additional file 4 — Characterization of microsatellites in the genome and EST sequences of the placozoan Trichoplax adhaerens. [file 1745-6150-5-18-S4.PDF]

Characterization of microsatellites in the genome and EST sequences of the placozoan *Trichoplax adhaerens*

	Mononucleotide		Dinucleotide		Trinucleotide		Tetranucleotide		Pentanucleotide		Hexanucleotide	
	Genome	EST	Genome	EST	Genome	EST	Genome	EST	Genome	EST	Genome	EST
	A [1](274[2])	A (273)	AT (1338)	AT (52)	AAT (605)	AAG (309)	AATT (252)	AATT (10)	ACAGT (5624)	ACAGT (14)	AACCCT (53)	ACGTCC (12)
	C (193)	C (4)	AG (268)	AG (6)	ATC (210)	ACG (111)	AAAT (205)	ATAC (6)	AAAAT (302)	AAAAC (7)	AAAATT (33)	AACGTG (2)
			AC (52)		AAG (114)	AAT (15)	AATC (99)	ACGC (2)	AAATT (287)	AAAAT (6)	AAAAAG (19)	AAAAAT (2)
					AAC (96)	ACC (11)	AAAG (42)	AGCC (2)	AATAT (136)	AAATT (4)	AAAAAT (19)	ATCACC (1)
					ACT (57)	CCG (7)	ATAG (36)	ACTG (2)	ACCGT (95)	ACCGT (4)	AATTAT (19)	AAAAAG (1)
					AGC (29)	ATC (7)	ATAC (24)	AAAT (1)	ACTCT (61)	AATAC (2)	ATATAC (13)	
					ACC (21)	AAC (5)	AATG (21)		AACCG (58)	AATTC (2)	ACTGGT (11)	
					AGG (17)		ACTG (18)		AATTC (54)	AACAT (2)	AATACT (11)	
					ACG (16)		AACT (13)		AAAAG (41)	ACCCC (2)	AATAGT (9)	
							ACAG (10)		ATACT (41)	AAAAG (2)	AAATTT (9)	
							AAGT (10)		AATAC (34)	ACTCT (1)	AAGATG (8)	
							AAAC (8)		AATAG (32)		AAAATC (7)	
							AAGG (7)		AAATG (31)		AAATAT (7)	
							ACCG (4)		AAATC (29)		ATATAG (7)	
							AGGG (4)		ACTGG (26)		AGCCTG (7)	
							ATCG (4)		AAACT (24)		ATCGTC (6)	
							ATCC (4)		ACCGG (23)		AACAGC (5)	
							AGCT (3)		ACCAG (20)		AATAAG (5)	
							ACTC (3)		AAAGT (20)		AAATAG (4)	
							AGCC (3)		AATCT (19)		ACTAGT (4)	
							AACC (2)		AATGT (19)		AAATTG (4)	
							ACCT (2)		ATATC (19)		ACTGCT (3)	
							AGCG (2)		AAGCT (16)		AACTAT (3)	
							AAGC (2)		AATCC (13)		AATTAG (3)	
							ATGC (2)		AATGC (12)		AAATTC (3)	
							AACG (1)		AACTG (12)		AATTAC (3)	
									ATAGG (10)		AATGCC (3)	
									AAGTG (10)		AAATGC (3)	
									AACAT (10)		AATAAC (3)	
									ACACT (9)		ATCAGC (3)	
									AAAAC (9)		AATATC (3)	
									AACTT (9)		AGCAGG (3)	
									AAGAT (7)		AATGAT (2)	
									ACAGC (6)		AAATAC (2)	
									AGGCC (6)		AAACAG (2)	
									ATGAC (6)		AAGTAG (2)	
									AAAGG (6)		AAAATG (2)	
									AAGGC (6)		AAATCG (2)	
									ATAGC (5)		AGAGGC (2)	
									ACTAG (5)		AATATG (2)	

									AATCG (5)		ACCTCG (2)	
									ATACC (4)		ATCAGG (2)	
									AAGAC (4)		AAGAGG (2)	
									AACGG (4)		AATATT (2)	
									ATCCG (4)		ATCTCG (2)	
									ACAGG (4)		AAAAGG (2)	
									AAGAG (4)		ACCGGC (2)	
									AAAGC (4)		AAAAGT (2)	
									AATGG (3)		AACCTG (2)	
									AACCT (3)		AATCTT (2)	
									AACGC (3)		ACCGGT (2)	
									ATCAG (2)		ATCCGG (1)	
									AAGCC (2)		ATCTAG (1)	
									ACGCT (2)		ATGTAC (1)	
									AGGGG (2)		ATAGTG (1)	
									AGCCT (2)		AACTCC (1)	
									ATACG (2)		AACAGT (1)	
									ACCTC (2)		ATACTG (1)	
									ACTCG (2)		AACATC (1)	
									AAACC (2)		ACCTAG (1)	
									ACGTC (2)		ATCCTC (1)	
									AACAC (2)		AATAGG (1)	
									ACGGC (2)		AAGTAC (1)	
									AAGTC (1)		ATACAC (1)	
									ACCCT (1)		AGCTGC (1)	
									AGCCG (1)		ATACTC (1)	
									ATCGC (1)		ACCTCC (1)	
									AACGT (1)		AATCGT (1)	
									ATCAC (1)		ACTGCC (1)	
									AGCTC (1)		AAGAGC (1)	
									AACTC (1)		AACGAC (1)	
									ACTCC (1)		AAGACT (1)	
									AACAG (1)		AAATCT (1)	
									ACCGC (1)		ACCTCT (1)	
									AAGGT (1)		AAACAC (1)	
									AGAGG (1)		ATATGC (1)	
									ACGGG (1)		AAAAGC (1)	
									AGGGC (1)		AAATGT (1)	
											ACTAGC (1)	
											AAGGAG (1)	
											AAAACG (1)	
											AACCAG (1)	
											AACCGG (1)	
											ACGTCC (1)	
											AGCTCC (1)	

											ATAGCG (1)	
											ACCGAG (1)	
											ATCACG (1)	
											AATCTG (1)	
											ACGAGG (1)	
											AAATGG (1)	
											ACTCGT (1)	
											AACGAT (1)	
											ACAGGC (1)	
											ACTCGC (1)	
											AATCAG (1)	
											ACTACC (1)	
											AACTAC (1)	
											ATAGGC (1)	
											AACTTG (1)	
											AAACAT (1)	
											AAAGAT (1)	
											AGAGCG (1)	
											AACGTG (1)	
											AATCCT (1)	
											AATAGC (1)	
											AAGCTG (1)	
											ATAGAG (1)	
											AAGCAG (1)	
											ACTACG (1)	
											AATGCT (1)	
											ACGCAG (1)	
											AAAGTC (1)	
Total	467 (4.0%[3])	277 (31.2%)	1658 (14.2%)	58 (6.5%)	1165 (10.0%)	465 (52.4%)	781 (6.7%)	23 (2.6%)	7233 (61.8%)	46 (5.2%)	393 (3.4%)	18 (2.0%)

1. Repeat motif type; 2. Total number of a given motif type; 3. Percentage of a given motif type in the genome- or EST-derived microsatellites.
