# Supplementary figures and images for: Genome-wide analysis of transposable elements and tandem repeats in the compact placozoan genome
Source: Biol Direct. 2010 Apr 15;5:18. doi: 10.1186/1745-6150-5-18 (PMC2871265; doi:10.1186/1745-6150-5-18)

The distribution of abundance (a) and average copy number (b) in minisatellites.

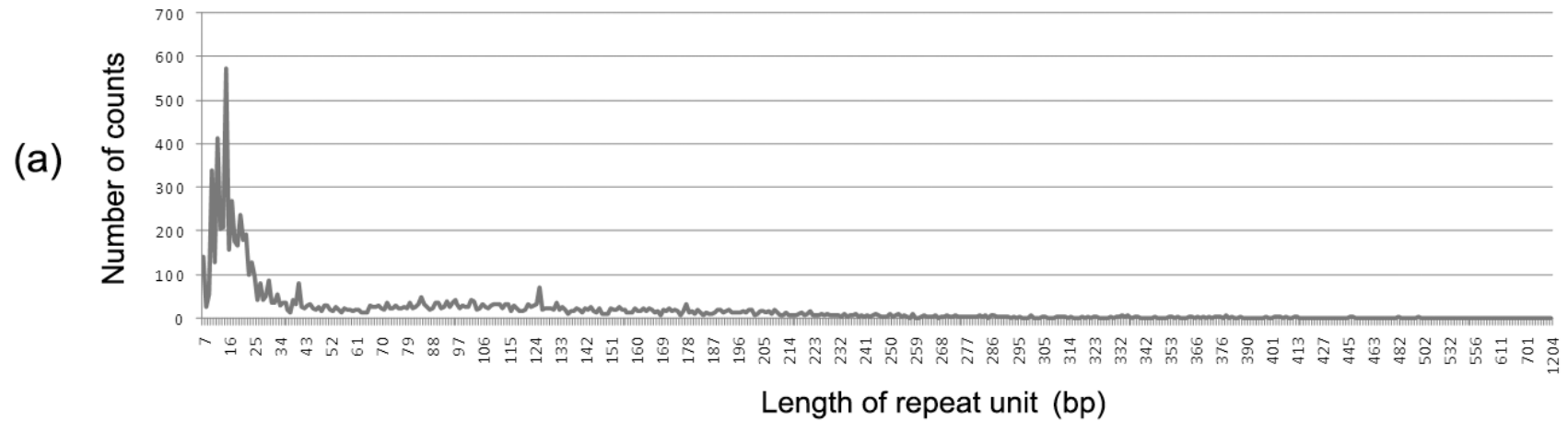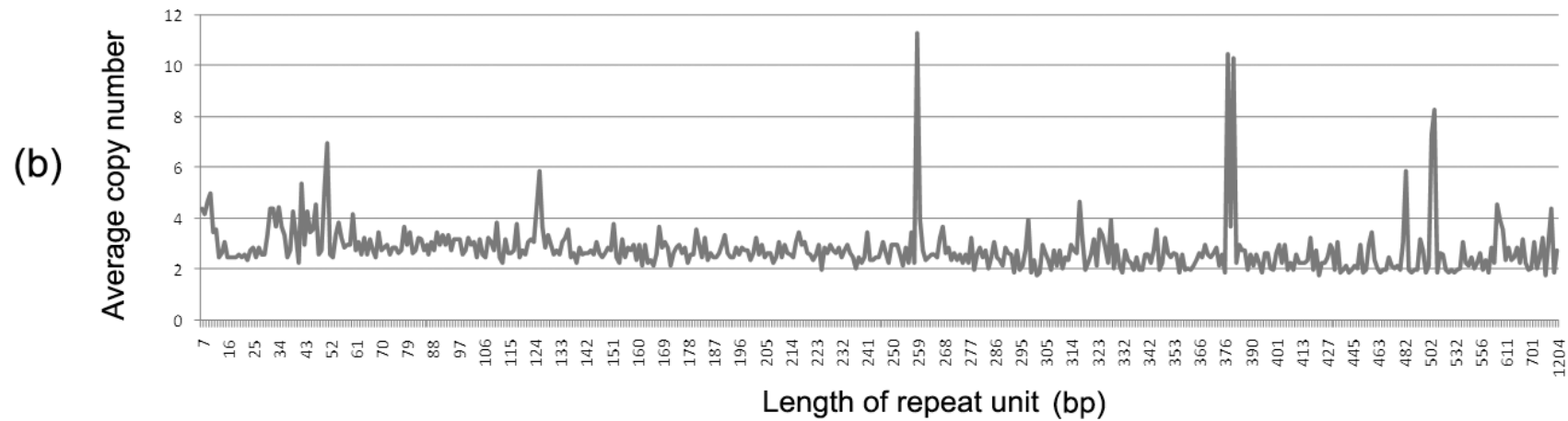

Supplement: Additional file 6 — The distribution of abundance and average copy number in minisatellites. [file 1745-6150-5-18-S6.PDF]
